# Colorectal cancer screening awareness among physicians in Greece

**DOI:** 10.1186/1471-230X-6-18

**Published:** 2006-06-06

**Authors:** Apostolos Xilomenos, Davide Mauri, Konstantinos Kamposioras, Athanasia Gkinosati, Georgios Zacharias, Varvara Sidiropoulou, Panagiotis Papadopoulos, Georgios Chatzimichalis, Vassilis Golfinopoulos, Christina Peponi

**Affiliations:** 1 Panhellenic Association for Continual Medical Research (PACMeR) Sections of Public-Health, 28, Karolou st, 10438 Athens, Greece; 2 Department of Medical Oncology, Ioannina University Hospital, 45500, Ioannina, Greece

## Abstract

**Background:**

Data comparison between SEER and EUROCARE database provided evidence that colorectal cancer survival in USA is higher than in European countries. Since adjustment for stage at diagnosis markedly reduces the survival differences, a screening bias was hypothesized. Considering the important role of primary care in screening activities, the purpose of the study was to investigate the colorectal cancer screening awareness among Hellenic physicians.

**Methods:**

211 primary care physicians were surveyed by mean of a self-reported prescription-habits questionnaire. Both physicians' colorectal cancer screening behaviors and colorectal cancer screening recommendations during usual check-up visits were analyzed.

**Results:**

Only 50% of physicians were found to recommend screening for colorectal cancer during usual check-up visits, and only 25% prescribed cost-effective procedures. The percentage of physicians recommending stool occult blood test and sigmoidoscopy was 24% and 4% respectively. Only 48% and 23% of physicians recognized a cancer screening value for stool occult blood test and sigmoidoscopy. Colorectal screening recommendations were statistically lower among physicians aged 30 or less (p = 0.012). No differences were found when gender, level and type of specialization were analyzed, even though specialists in general practice showed a trend for better prescription (p = 0.054).

**Conclusion:**

Contemporary recommendations for colorectal cancer screening are not followed by implementation in primary care setting. Education on presymptomatic control and screening practice monitoring are required if primary care is to make a major impact on colorectal cancer mortality.

## Background

Data comparison between EUROCARE and SEER database provided evidence that colorectal cancer survival in United States of America is higher than in European countries [1]. Survival differences were maintained irrespectively of which European Nation was compared, and were much higher when Eastern European countries were considered [1]. Correction for stage at diagnosis consistently reduced survival differences and the reduction was substantially unrelated to the European geographic area analyzed [2]. The presence of a diagnostic colorectal cancer screening bias was therefore hypothesized since early diagnostic procedures might be much less available in Europe than in USA [2]. Deficiencies in European colorectal cancer screening guideline implementation, and inadequacy of screening test advising in primary care setting were highlighted in a recent systematic review of literature, but only data from Italian and French physicians were available for the USA vs European data comparison [3].

Colorectal cancer screening survival benefit had been strongly documented by randomized-controlled trials [4-8] and a meta-analysis [9]. In this setting, time to diagnosis and, more precisely, stage at surgery, play the major role for patient outcome. In addition to detecting early stage cancers, screening can detect pre-cancerous polyps as well, which can be removed, thereby preventing cancer. Colorectal cancer screening is therefore strongly recommended. Currently guidelines generally include: stool occult blood test (SOBT) yearly and sigmoidoscopy every 3–5 years, both beginning at the age 50 [10-13]. There are two major ways to implement these recommendations: organized screening programs principally endorsed with active invitations by the national health services; and opportunistic screening recommendation by general practitioners. Despite organised screening programmes are based on a more coherent structure, offering a standardised system of care and are at a recognized better level of evidence, National CCS (colorectal cancer screening) programs are actually at the beginning in European countries, and the ongoing pivotal experiences such as the Italian (region-wide programs), UK and Finnish (started in 2000, 2001 and 2004 respectively) are too recent to impact on survival [14-16]. Consequently, up to date opportunistic screening implementation in European primary care setting was mandatory. Indeed, physicians involved in primary care both implement programs with active invitation [17] and recommend screening tests where invitation programs are lacking. This is of particular importance in Greece where organized colorectal cancer screening programs are totally absent.

Since primary care physicians have a key role in screening practice, and considering that little is known about their screening recommendations in Europe [3], we surveyed a random sample of Hellenic physicians employed in primary care activities. Both physicians' colorectal cancer screening behaviors and colorectal cancer screening recommendations during usual check-up visits were analyzed.

## Methods

### The Hellenic trial

This study is part of a large ongoing program of research on cancer screening practice in Greece, which is organized by the PACMeR (Panhellenic Association for Continual Medical Research). The study's aim is to indicate the current rate of cancer screening among the Hellenic adult population and to identify possible barriers to early diagnosis. In PACMeR_03 trial a medical questionnaire for face-to-face interview was employed to investigate primary care physicians' screening prescriptions habits. The project was approved by PACMReR's Scientific Committee and conformed to the ethical guidelines of the 1975 Declaration of Helsinki.

#### Enrollment

From August 2001 to December 2002, 600 medical doctors employed in primary care activities were recruited from medical lists of 14 health centers, 16 general hospital and 31 rural ambulatory departments in 9 Hellenic provinces (Achaia, Attikis, Chania, Cephalonia, Drama, Etolocarnania, Evro, Lesbo, Serres) and were invited to answer the prescription-habits questionnaire in a self-reported and nominal form during a face to face interview with a PACMeR physician. Personal data privacy was warranted for all physicians who refused to enter the trial.

Two hundred eleven (211) physicians agreed to participate: 105 male, 94 female, 12 did not declare gender (privacy was guaranteed in spite of the face-to-face nature of the survey). The mean age of physicians involved in the study was 34 (24 – 62 years old); 31 were specialists in General Medical Practice (GMP), 22 trainees in GMP, 25 Internists, 53 trainees in Internal Medicine (employed in internal medicine ambulatory department with activities overlapping primary care), 78 unspecialized physicians (employed in the compulsory rural primary care medical service), 1 who did not answer this item.

#### Medical questionnaire

Physicians were invited to answer two main questions: one for usual screening practice and one for specifically indicated cancer screening practice.

1^st ^Question: "When a patient older than 50 years old comes to your office and asks for a general check up what do you recommend?"

2^nd ^Question: "Which of the following examinations do you think should be prescribed to people older than 50 for cancer screening practice?"

The medical questionnaire contained a board of multiple choices for 58 possible (related or unrelated) screening procedures (i.e. digital rectal examination, chest radiography, Papanicolaou smear, urinalysis, urinary culture, prostate specific antigen).

For the 2^nd ^Question the prescription frequency (monthly, twice a year, yearly, every 2 years, 3 years, 5 years and no prescription) should have been specified. Data obtained were further analyzed for sigmoidoscopy, stool occult blood test and digital rectal examination.

Due to the phrasing of the two questions, and considering that DRE may be implemented for both colorectal and prostate cancer screening, the determination of the implemented proportion of the test in each setting was not possible, and related analysis should be therefore considered a secondary outcome.

#### Subgroups analysis and statistics

Since it might be guessed that trainees and recently trained physicians would be more likely to be aware of evidence-based and cost-effectiveness studies, screening habits were further analyzed for physicians' subgroups by age, sex, level and type of specialization. Since the digital rectal examination is not a cost effective screening procedure, we considered its prescription of not value [18]. Colorectal cancer screening habits were therefore divided in two categories: no prescription OR prescription of at least one documented cost-effective test (sigmoidoscopy or stool occult blood test) at any frequency. Statistical analysis was performed using Pearson's Chi-square test and Fisher's exact test.

## Results

We found that the 50% (106/211) of physicians recommended colorectal cancer screening during usual check-up visits. After exclusion of non-cost-effective screening procedure (digital rectal examination) from the analysis, the percentage of physicians recommending colorectal screening (stool occult blood test and/or sigmoidoscopy) shrank to 25% (52/211). Only the 3% (7/211) of physicians was found to recommend both sigmoidoscopy and fecal occult blood test; while the 1% (2/211) advised sigmoidoscopy alone and the 20% (43/211) fecal occult blood test alone.

When physicians were specifically asked about which examinations they think should be prescribed to people older than 50 for cancer screening practice, only the 77% recognized a value for colorectal cancer screening tests (any prescription considered: stool occult blood test and/or sigmoidoscopy and/or digital rectal examination). This percentage dropped to 53% (112/211) when only cost-effective procedures were considered (stool occult blood test and/or sigmoidoscopy). In this setting, we found that 18% (39/211) of physicians think that both sigmoidoscopy and stool occult blood test should be prescribed for cancer screening activities, while 4% (9/211) recommended sigmoidoscopy alone, and 30% (64/211) for stool occult blood test alone. Frequencies by which physicians prescribe the abovementioned tests for screening purposes are reported in Table 1.

### Subgroups analysis

The colorectal cancer screening recommendations rate during usual check-up visits were markedly higher among specialists in general medical practice (45.2%) even though the difference was not statistically significant (exact p = 0.054). It was quite homogeneous among the other specialization subgroups (trainees in general medical practice 18.2%, internists 20%, trainees in internal medicine 22,6% and doctors employed in the post-law compulsory rural primary care medical service 20,5%). Colorectal cancer screening recommendation rate during usual check-up visits was statistically lower among physicians aged 30 or less versus older ones (p = 0.012).

When cancer-screening behaviors were considered, we found no belief differences among physicians' subgroups for any of the analyzed parameters: specialization (exact p = 0.749), age (p = 0.057), gender (p = 0.191).

## Discussion

Little is known in peer-reviewed literature about colorectal cancer screening recommendations in European primary care. Up to date only 4 surveys had been reported (three from France and one from Italy) [19-23] and relative results had been recently analyzed in a systematic review of literature [3]. Present study is therefore the first report from Greece and from a third European nation.

In spite of the documented survival benefit from colorectal cancer screening [4-9], regardless of its recommendation by health authorities [10-13], and in sharp contrast with the high rate of CCS recommendation by US physicians [24], the rate of CCS implementation was not satisfactory in any of the European studies but one [21] and particularly in the present survey (table 2). Indeed in Ganry study (2004)[21] a significant higher rate of CCS advising (95%) was observed in comparison with previous French reports (1996, 2003)[19,20]; this may be explained by the fact that time from guideline implementation (1998 for France)[25], related medical education and putting guidelines in to practice are sequentially time dependent processes. In our trial 46% of physicians screened their healthy patients with digital rectal examination during regular general practice activities. This percentage was notably higher (68%) when cancer-screening purpose was considered. Surprisingly, recommendation of this not evidence-based test had been previously reported among French physicians [19,21]. Indeed, despite the "myth" of digital-rectal examination is alive among physicians, its implementation as screening procedure is not cost effective [18] and therefore not recommended by major authorities [10-13,26,27].

We further observed that a large proportion of physicians did not consider cancer-screening practice as a part of the periodic health examination (only 50% of physicians advise it during usual check-up visits, while the 77% believe it should be prescribed in case of cancer screening activities). The more discomforting finding in our trial was that only the 25% of the surveyed physicians recommended evidence-based screening tests during usual check-up visits.

These results might be in part explained by the actual composition of the Hellenic health system. Indeed, to date primary care services were mainly guaranteed by non-specialized physicians employed in the post law compulsory medical service (for free service supported by the Health system) and by private family physicians (for fee services); and secondarily by internal medicine ambulatory division of general hospital and by professional funds (for free services). Thus, non-specialized young physicians are the key of the actual Hellenic primary care system. Therefore, the low rate of screening recommendation observed should not be surprising and explains partially why in our study colorectal screening recommendations were statistically lower among physicians aged 30 or less. In fact we found that specialists in general practice showed a trend for better prescription (p = 0.054).

Fortunately, the newborn sanitary system is progressively substituting the unspecialized post law physicians with specialized GPs. The whole primary care composition, as well as services provided, may consequently radically change in next 5–10 years. We therefore hope that a specialized system will provide both the hopeful changes and a cost-effective screening coverage.

Anyway, our study presents many limitations and may not be applicable to the whole population of primary care physicians. Firstly we have data of only 211 physicians. Secondly, practices of responders may be systematically different from those of non-responders; a high rate of non-responders is anyhow common in this kind of cross-sectional survey [28-30]. Finally, lack in the availability of dedicated local endoscopic services may discourage physicians in recommending screening test; something that was not assessed in our study.

The Hellenic need of a new redefinition for the primary care setting should not be considered a local phenomenon. Poorly defined role of primary care physicians in screening services delivery and the need of a new re-definition of primary care activities have been evidenced even in a recent survey of English practitioners [31]; and screening data coming from French and Italian surveys were quite discouraging too [19-23]. This may be in part attributed to the history of guidelines implementation in both single Nation and European setting. Indeed, the date of guideline release is of a great importance since their dissemination, their cultural re-elaboration and the process of putting them into practice are time-dependent processes [3]. Actually in Greece there are not national guidelines for colorectal cancer screening and European guidelines were first time implemented only in 2003 [10].

## Conclusion

Since early diagnosis of colorectal cancer and stage of disease at surgery are crucial for patients' survival, we conclude that a huge work has to be done in Hellenic and, more widely, in European primary care setting if screening is to make a major impact on colorectal cancer mortality. Need for national guideline implementation and continual medical education are imperatively highlighted.

## Competing interests

The author(s) declare that they have no competing interests.

## Authors' contributions

AX: Main coordinator, he was actively involved in the discussion of the project & study planning, and manuscript writing. DM: He was actively involved in the discussion of the project, realization of the draft & study planning, review of data abstraction and manuscript writing. KK: was actively involved in the discussion of the project & study planning, and in the data collection during the survey. AG: She was involved in study planning and she was responsible for data collection in Crete Island. She was still actively involved in the discussion of the project and manuscript. GZ: He was involved in the study planning and was responsible for data collection in the wide area of Attika. He was still actively involved in the discussion of the project and manuscript. V S: She was involved in the study planning and was responsible for data collection in northern Greece. She was still actively involved in the discussion of the project and manuscript. PP: He was involved in the study planning and was responsible for data collection. He was still actively involved in the discussion of the project and manuscript. GC: He was involved in the study planning, in data collection and in the discussion of the results. VG: He was actively involved in the discussion of the project, realization of the draft, discussion of the outcomes, reviewing and formatting the manuscript. CP: Main data-manager. She was involved in the study planning and was responsible for data collection in the northwestern part of Greece. She was responsible of data entering in the database. She was involved in manuscript discussion.

## Pre-publication history

The pre-publication history for this paper can be accessed here:



## Figures and Tables

**Table 1:** Hellenic PCPhs' recommendations of colorectal tests for cancer screening activities:

**Screening tests**	**twice a year %**	**yearly %**	**every 2 years %**	**every 3 years %**	**every 5 years %**	**Useful undefined frequency %**	**Not useful %**
**SIG**	0	6	3	6	7	1	77
**SOBT**	3	35	6	1	1	1	52
**DRE**	6	55	4	1	1	1	32

**Abbreviations used**:

PCPhs primary care physicians

SIG sigmoidoscopy

SOBT stool occult blood test

DRE digital rectal examination

% percentage

**Table 2:** European studies indexing primary care physicians' recommendations of colorectal cancer screening during general practice activities.

**Author**	**Physicians analyzed**	**CCS %**	**SIG %**	**SOBT %**	**COL %**	**DRE %**	**Country**
PACMeR^##^	211	50	4	24	NA	46	Greece
Garcia Giannoli ^19^	163	65	0	42	0	38	France
Denis B^20^	374	66	0^#^	44	6	NA	France
Ganry ^21^	480	95	NA	83	10	17	France
Federici ^22^	699	78	55	64	48	NA	Italy

**Abbreviations used**:

^## ^Presented report

^# ^Data obtained from personal communication with the author

CCS Percentage of physicians recommending colorectal cancer screening practice

SIG sigmoidoscopy

SOBT stool occult blood test

COL colonoscopy

DRE digital rectal examination

% percentage

NA data not assessed
